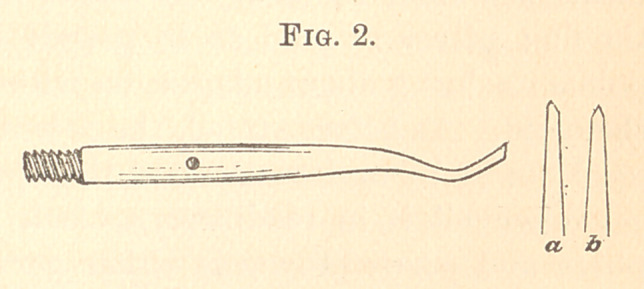# The Fixation of Dental Matrices and the Packing of Gold at the Cervical Portion of the Cavities

**Published:** 1892-08

**Authors:** Louis Jack

**Affiliations:** Philadelphia


### International Dental Journal.

VOL. XIII. AUGUST, 1892. No. 8.

### Original Communications.[1]

### THE FIXATION OF DENTAL MATRICES AND THE PACKING OF GOLD AT THE CERVICAL PORTION OF THE CAVITIES.[2]

BY DR. LOUIS JACK, PHILADELPHIA.

THE consideration of these important features connected with the use of dental matrices is entertained for the reason that the difficulties which have usually been encountered have generally pertained to these two procedures.

The general purposes of the matrix are to make less difficult the formation of the proper contour of proximate surfaces, to effect the placement of the gold in exact adaptation to the cervical wall with sufficient solidity, and to secure these ends without injury to the structure of the teeth.

This appliance greatly facilitates the packing of cavities on the distal proximate surfaces of molars and bicuspids, for which surface it is more especially intended as being particularly well adapted to these positions.

With the increased deftness which grows out of experience with this aid, it becomes frequently an important assistance to the performance of many mesial proximate cavities.

---

[1] The editor and publishers are not responsible for the views of authors of papers published in this department, nor for any claim to novelty, or otherwise, that may be made by them. No papers will be received for this department that have appeared in any other journal published in the country.

[2] Read at a meeting of the American Academy of Dental Science, held at Boston, March 2, 1892.

It is not necessary to dwell upon the details of the preparation of the carious cavity. Let it suffice to state that when the cavity is situated on the distal proximate surface of the molars, the opening of the orifice should include a large portion of the masticating plate of enamel; that the walls should be undercut at the inner and outer margins, so far as may be done without weakening the strength of the borders; that it is not necessary the undercutting should extend entirely to the cervical margin; that the cervical wall should be transverse to the axis of the tooth and be formed without undercuts or retaining pits; that the margins at all points should be countersunk, but to a much less degree at the cervix than at the lateral margins. It is also important the margins of the cavity be polished to facilitate the movement of the gold by the removal of the friction. When the cavities are situated nearer the front of the mouth, the removal of the masticating wall becomes only so far necessary as to facilitate the introduction of the gold and to eliminate so much of this plate as may be subject to impairment by the force of mastication.

For the ordinary cases of teeth which are not impaired by fracture or by the loss of structure by previous extensive cutting, the forms of matrices which I use are the depressed and those of plane surfaces bent and formed to meet the exigencies of the case in hand.

Where the depressed form is used the depression should correspond in size to the dimensions of the cavity, and its edge should extend a short distance above the cervical margin. This form of matrix is better adapted when the margins of the cavity have been so far weakened by carious action as to necessitate trimming away a portion of the buccal wall, and where for reasons beyond control the teeth are considerably separated.

The plain matrix is better adapted when the separation between the teeth is slight. It should be stated that in all cases it is necessary for the securement of correct results that the preliminary step in each case should be the separation of the teeth by pressure; this is to enable the parts to be properly finished, and to have the gold of the one tooth come into contact with the gold of the adjoining tooth at the natural points of contact of the proximate surfaces of the teeth. These preliminaries enable the consideration to be made of the fixation of the selected matrix.

The important requirements concerning this procedure are that the matrix shall be in contact with the cervical surface of the tooth as nearly throughout that margin as it is possible to have it; that the matrix shall be in contact with the lingual margin of the cavity, and be in near contact with the palatal margin below the cervical portion.[1] This feature of the fixation of the dental matrix is at some difference with the views of some others concerning this point, but I hold that to impact the gold by the percussive force necessary to produce contact and solidity at the cervical border the line of junction should be a close one. This hypothesis has been borne out by considerable experience with the two conditions of proximity and apparent contact. The securement of contact at these two points of the margin is attained by the peculiar form of the wedges to be used. The one for the cervical border is a double wedge. The sectional dimensions depend upon the space at the cervix and somewhat upon the general area of the cavity, and its length upon the lateral size of the tooth. This wedge, after being dipped in Sandarac varnish of the consistency of syrup (any excess of the varnish being removed), is forced into the space by pressure. If of proper size it should appear at the inner margin, and extend beyond the outer surface far enough to allow a grasp to be made for the removal. The wedge for the palatal margin is also somewhat of double wedged form, but it is not sharp at the point.

At *a* (Fig. 1) the piece appears as a simple double wedge having an even width, but becoming thinner on the back at the end, to be applied at the cervix. At *b* the end is cut in the form presented. It should be of size to correspond to the space between the matrix and the tooth adjacent to the subject of the operation. When the tooth is much rounded the face of the wedge often requires to be hollowed out with a gouge, the better to adapt it to the space. This wedge is varnished, put in place, and forced into position by an instrument pushed against its thick edge at the upper part. The object of the mitre-like form at the end now appears, as this surface corresponds to the line of the cervix. In some cases this end will be apposed against the cervical wedge; in other cases it will pass between the adjacent tooth and the cervical wedge. In either case it makes this portion of the matrix secure and permits the contour to be effected.

Fig. 1.

[1] In using the words "above" and "below" in relation to the cavity, the tooth is considered out of its topographical position, the apex being considered above and the corona below.

It may appear a small matter to dwell with so much detail upon so apparently trifling a matter as a wedge for this purpose, and yet it is upon the absence of definite knowledge of a correctly mechanical fixation of the matrix that has prevented many operators from securing the great benefits which are to be found in the employment of matrices. It is, I believe, the absence of the thoughtful application of these simple means which has led to the invention of complicated and, I believe, less effective methods for fixation. It will be perceived by the accompanying casts the adaptation of this method to which attention is directed.

### THE FILLING OF THE CERVICAL PORTION.

The chief elements connected with this feature of the subject concern the character of the gold, the form of the pieces of gold, the form of the instruments best adapted, and the method of applying the instruments. Each of these considerations is important, and a disregard of the qualifications of the filling material or of any of the essential procedures may defeat the ends to be secured by this system of filling cavities.

The gold to be employed for the upper half at least of the space should be non-cohesive and soft; that is, it should be incapable of being rendered cohesive by heat, and should remain soft and lead-like during the manipulation to which it is subjected. The gold I have found to fulfil this requirement more nearly than any others which I have used is the so-called soft gold of Morgan & Hastings. The soft gold of Abbey & Sons is also well adapted, but each one should make his own tests of the quality in this respect of the gold to be used.

The reason that gold of a cohesive nature should not be employed is that the portion in immediate or close relation to the instrument may become consolidated and bridge before the point in such a manner as not to permit the force to be expended to the ultimate portion of each piece of gold which is being moulded into position and undergoing consolidation.

### THE FORM OF THE PIECES.

That form which will be found best adapted in the larger cavities is produced by folding a tape into a block. The tape is formed by folding from one-third to a whole sheet of No. 4 or No. 5 three times, thus producing a tape of eight layers. The division of the sheet is dependent upon the dimensions of the space and somewhat by the strength of the walls. The folding of the tape into blocks is also, as to form and number of turns made, dependent again upon the size of the cavity. These variations can only be learned by the study and experiment which each one should make for his own guidance, and for which no theoretic rule can be defined.

THE FORM OF THE INSTRUMENTS.

It is obvious the shape of the plugging instruments to be used in this system of stopping cavities must be different from those which are applicable to the ordinary methods. The bends of the shank should be such that the points may reach all parts of the cavity, and the form of the ultimate point is required to be that which will be best calculated to produce adaptation and solidity of the gold at the lines of apposition of the matrix and the walls of the cavity, and also should be so shaped that by slight rotation of the instrument on its axis the point may reach into the retaining grooves, to carry on the consolidation of the gold at this point.

Fig. 2 represents the shape which I have found to be more nearly universal for this purpose than any others, and, as will be seen, differs only in the form of the ultimate point first described and published as a matrix plugger.

FIG. 2.

It will be observed the ultimate point is not flat and transverse to the axis, but that it is ovoid and slightly bevelled to give the extreme edge a little to one side. This necessitates the points to be in pairs, but for the general packing the points are transverse laterally and slightly ovoid. In the use of the instruments the edge is held at an angle to the marginal line, to prevent the point being driven between the matrix and the margin of the cavity; by this caution and slight tilting movements deft use of the instrument is quickly acquired.

When these are made of several sizes and of slightly varying bends they will meet the requirements of any case which may occur. The serrations are made very fine, and are necessary only to inhibit the instrument cutting the foil and to maintain a frosted surface, which prevents too easy sliding of the gold.

THE PACKING OF THE CERVICAL PART OF THE SPACE.

The chief ends in view in the use of matrices are the facilitation of the procedure of packing the gold and the perfect adaptation of the gold to the cervical wall. The difficulties attending the adaptation of gold-foil to the cervical walls of distal cavities, as applied in the ordinary methods, are caused partly by the posterior position, and partly by the physical tendency of gold to draw away from the margins during the packing process. This tendency pertains to all forms and kinds of gold used. When the cases are upon accessible surfaces this disposition is overcome by leverage movements of the filling instruments, and by the employment of foot points. On the distal surfaces, when the spaces are comparatively small, the opportunity for leverage does not exist, and the foot point is not convenient of application.

There is another important advantage this method possesses. When distal cavities are filled by the ordinary plans, it is necessary either to produce deep retaining grooves near the cervical region or to make retaining pits at this part. Each of these have serious objections; the first weakens the lateral walls, and the latter impairs the vitality of the adjacent portion of dentine, and also in many instances imperils the safety of the pulp. These deep retaining means are then necessary to insure that degree of securement of the first pieces of gold as to permit the malleting with the foot points to bring about adaptation without causing displacement of the gold. As before stated, the retention when the matrix is used need not be by undercutting at the upper part of the lateral walls.

When all is in readiness, a block of the gold is seized in the pliers and carried towards either cervical lateral position in such manner as to direct its end towards the countersink. It is then secured by the use of any suitable plugger which will fix it in position. The opposite aspect of the cavity is treated in the same manner when the special pluggers are used to force by percussion the blocks into adaptation with the margins. A third piece is next adjusted between the two. When this is fixed in position careful adaptation may be effected by going over the whole surface, particular attention being directed to secure contact along the line of junction of the matrix with the walls.

The size of the selected blocks should be such that they will by their dimensions assist by the friction in holding their place. As the gold should be soft and yielding, there will be no impediment by the size unless this should be out of reasonable relation to the dimension of the space to be filled. When the first layer is completed others may be made seriatim until the upper third or half of the cavity is filled, when the major part of the balance should be completed with cohesive gold. For this part my reliance has been upon No. 20. The amount of force required to effect the consolidation is not great, and as the gold is confined by the limitation of the space it can easily be brought into adaptation. When the automatic plugger can be used, the force of the short blows of this is sufficient. The Bonwill mechanical mallet is also well adapted in its force to effect the consolidation, and is peculiarly well suited to mesial surfaces.

I have in some detail thus described a procedure which, in my own experience and that of others, has proved of great value, not only as a means of effecting good results, but as a measure which facilitates the completion of the most difficult class of operations which we are called upon to perform.

## Figures and Tables

**Fig. 1. f1:**
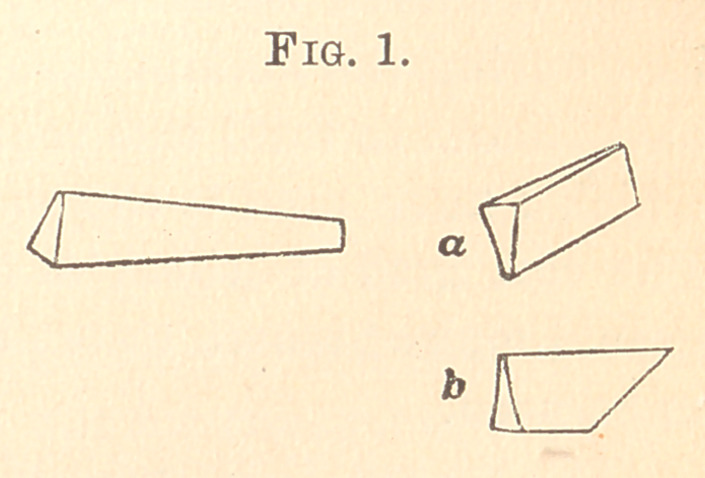


**Fig. 2. f2:**